# Two new combinations in *Oreocharis* (Gesneriaceae) based on morphological, molecular and cytological evidence

**DOI:** 10.3897/phytokeys.157.32609

**Published:** 2020-08-26

**Authors:** Li-Hua Yang, Fang Wen, Hang-Hui Kong, Zhi-Xia Sun, Lan-Ying Su, Ming Kang

**Affiliations:** 1 Key Laboratory of Plant Resources Conservation and Sustainable Utilization, South China Botanical Garden, Chinese Academy of Sciences, Guangzhou, CN-510650, China Chinese Academy of Sciences Guangzhou China; 2 Guangxi Key Laboratory of Plant Conservation and Restoration Ecology in Karst Terrain, Guangxi Institute of Botany, Guangxi Zhuang Autonomous Region and Chinese Academy of Sciences, Guilin, CN-541006, China Guangxi Institute of Botany Guilin China; 3 University of Chinese Academy of Sciences, Yuquan Road, Shijingshan District, Beijing, CN-100049, China University of Chinese Academy of Sciences Beijing China; 4 College of Life Sciences, Guangxi Normal University, Guilin, CN-541004, China Guangxi Normal University Guilin China

**Keywords:** *Beccarinda baolianis*, *Boeica guileana*, ITS, *
Oreocharis
*, taxonomy, *trnL-F*

## Abstract

The newly-circumscribed genus *Oreocharis* is recently enlarged by incorporating ten other genera with high floral diversity. In this study, our morphological, molecular and cytological evidence supports our adding two species from other two different genera (*Boeica* and *Beccarinda*) to *Oreocharis*. The special corolla shape (campanulate or flat-faced) and related short filament of these two new combinations, *Oreocharis guileana* and *O. baolianis*, further enrich the diversity of floral characters of the enlarged *Oreocharis*. Meanwhile, some supplementary and amended descriptions of these two species are made here. Our morphological, molecular and geographical data indicate that *O. guileana* is related to *O. pilosopetiolata* to a certain extent. For *O. baolianis*, however, our current dataset does not allow conclusions on the species relationship within *Oreocharis*.

## Introduction

Recent phylogenetic studies on the Old World Gesneriaceae have greatly advanced our understanding of species relationships and generic delimitations in this plant group (Wang et al. 2010; Möller et al. 2011a). One of the typical cases is the re-circumscription of *Oreocharis* Bentham. Molecular phylogenetic analyses indicate that species of the original *Oreocharis* are phylogenetically intertwined with species of ten other small or monotypic genera (Möller et al. 2011b). After considering both the phylogenetic conclusion and the morphological evaluation, Möller et al. (2011b, 2014) formally transferred all species of nine genera (i.e. *Ancylostemon* Craib, *Bournea* Oliver, *Dayaoshania* Wang, *Deinocheilos* Wang, *Isometrum* Craib, *Opithandra* Burtt, *Paraisometrum* W.T. Wang, *Thamnocharis* Wang and *Tremacron* Craib) and rosette species of *Briggsia* Craib (except *B. longipes* and *B. mihieri*) to *Oreocharis*. This expansion not only made the *Oreocharis* a large genus within subfamily Didymocarpoideae (tribe Trichosporeae, subtribe Didymocarpinae) of the Gesneriaceae (Weber et al. 2013), but also made it one of the florally-diverse groups. Species of the newly-circumscribed *Oreocharis* all represent rosette herbaceous plants with spirally-arranged leaves, scapose inflorescences and loculicidally dehiscent capsules; however, their floral characters are extremely variable (Möller et al. 2011b). For instance, the characters corolla shape and colour, stamen number, placement of fertile stamens and anther coherence and shape are highly varied in this genus. The expansion of the morphological boundary of *Oreocharis* calls for reconsideration of the generic status of some problematic species; in particular, species whose generic placements are heavily based on flower characters, such as *Boeica guileana* B.L. Burtt and *Beccarinda baolianis* Q.W. Lin.

*Boeica guileana* was first described in 1977 by Burtt based on cultivated plants without description of mature fruits. The species has been regarded as endemic to Hong Kong for a long time (Burtt 1977; Wang et al. 1998; Li and Wang 2004); however, it was later discovered in Shenzhen, southern China (Wei et al. 2010). Based on corolla shape (short tube and spreading limb) and morphological resemblance to two existing species (*Boe. brachyandra* Ridl. and *Boe. nutans* Ridl.), Burtt (1977) assigned the plant to *Boeica* Clarke. However, *Boe. guileana* is distinctly different from most of other *Boeica* species by its rosette herbaceous habit, spirally-arranged leaves and longitudinally dehiscent anthers. Thus, in the original protologue, Burtt (1977) emphasised that the inclusion of *Boe. guileana* (S China) and two Peninsular Malaysian species (*Boe. brachyandra* and *Boe. nutans*) into *Boeica* is questionable. The generic status of all these three species is furthermore doubted by Weber and Skog (2007).

*Beccarinda baolianis* was newly described in 2016 from southern China, based on two specimens (Lin 2016). At present, only one population of this species can be found at its type locality (Lin 2016). Based on floral characters, such as the campanulate corolla and four stamens and capsule at a 90° angle to the pedicel, Lin (2016) assigned this species to *Beccarinda* Kuntze and compared it with *Be. minima* K.Y. Pan. However, our field observations found that the 90° angle between capsule and pedicel is not a stable character in the population. In addition, the inconspicuous rhizome and rosette habit of this species allow us to assume that it may only be distantly related to *Beccarinda*. Moreover, other important characters, i.e. the way of dehiscence in anthers and fruits, were not even described by Lin (2016). In the original protologue, Lin (2016) excluded this species from the original *Oreocharis* for its ovate anthers with apically confluent thecae, but this character does exist in the enlarged *Oreocharis*.

Summarising, the generic placements of *Boe. guileana* and *Be. baolianis* need reconsideration. Therefore, this study aims to clarify the generic status of these two species and discuss their species relationships in the enlarged *Oreocharis* using cytological, molecular and morphological analyses.

## Materials and methods

### Morphological observation

The previous descriptions of *Boe. guileana* and *Be. baolianis* were based on only one or two specimens from a single location; thus, the described characters do not reflect the whole range of variation and other characters were not described at all. For example, the dehiscent way of fruit of both of these two species had not been mentioned in the original protologue. To revise and amend the morphological descriptions and clarify the morphological affinities between these two species and its congeners, detailed morphological studies were made on living plants in the field (Shenzhen and Fujian, China) and on plants cultivated in greenhouses (South China Botanical Garden, SCBG and Gesneriad Conservation Center of China, GCCC). Furthermore, dried specimens were investigated. Checking of specimens was undertaken at IBSC, IBK, SZG and FAFU. Additionally, high-resolution images of specimens were critically checked using the web service of E (http://data.rbge.org.uk/search/herbarium/) and HK (http://www.herbarium.gov.hk/index.aspx).

### Molecular taxon sampling

In the recent classification of Gesneriaceae, *Boeica* and *Beccarinda* were deposited in subfamily Didymocarpoideae, tribe Trichosporeae, subtribe Leptoboeinae and the enlarged *Oreocharis* was deposited in subtribe Didymocarpinae (Weber et al. 2013). To verify the generic placements of *Boe. guileana* and *Be. baolianis*, we sampled a total of 82 ingroup samples, comprising almost all genera of the subtribe Leptoboeinae and 24 out of 34 genera in subtribe Didymocarpinae. Two species, *Rhynchoglossum obliquum* Blume and *Stauranthera grandifolia* Benth., were selected as outgroup, according to the phylogeny of the Old World Gesneriaceae (Möller et al. 2011a).

### DNA extraction, PCR and sequencing

The plastid *trnL-F* and nuclear ribosome internal transcribed spacer (ITS) sequences were used for the phylogenetic analysis. Most of these DNA sequences were acquired from GenBank and the sequences of six species, *Boe. guileana*, *Boe. ornithocephalantha* F. Wen, T.V. Do & Y.G. Wei (Wen et al. 2016), *Be. minima*, *Be. baolianis*, *Oreocharis pilosopetiolata* L.H. Yang & M. Kang (Yang et al. 2015) and O. benthamii var. reticulata Dunn, were newly amplified and sequenced in this study. DNA was extracted from dried leaves using a modified CTAB procedure described by Doyle and Doyle (1987). The polymerase chain reaction (PCR) amplification procedures and the PCR primers of these regions were described in Kong et al. (2017). Newly-amplified sequences were deposited in GenBank. The GenBank accession numbers used in this study are listed in Suppl. material 1.

### Sequence alignment and phylogenetic analysis

Sequence matrices of *trnL-F* and ITS were separately aligned using the programme MUSCLE implemented in the software MEGA7 (Kumar et al. 2016) with minor manual adjustments. A combined ITS and *trnL-F* matrix was generated using SequenceMatrix (Vaidya et al. 2011). The incongruence length difference test (Farris et al. 1994) was implemented in PAUP*4.0B10 (Swofford 2002) to assess potential congruence between ITS and *trnL-F*. Phylogenetic analyses were performed on a high-performance computer cluster available in the CIPRES Science Gateway 3.3 (www.phylo.org, Miller et al. 2015). Bayesian Inference (BI) in MRBAYES 3.2.6 (Ronquist et al. 2012) and Maximum Likelihood (ML) in RAxML8.2.10 (Stamatakis 2014) were calculated for each of *trnL-F*, ITS and the combined sequence matrix. Best-fitting models for MrBayes were obtained separately for *trnL-F* and ITS spacers through AIC in Smart Model Selection (SMS) by its web server (www.atgc-montpellier.fr, Lefort et al. 2017) and were GTR+G and GTR+I+G, respectively. A total of 10,000,000 generations were run in two independent analyses, each with four Markov Chain Monte Carlo (MCMC) chains. One tree was sampled every 1,000 generations and the first 2,000 trees discarded as burn-in. Posterior probabilities (PP) obtained from the analysis were used to indicate the credibility of various branches. The ML tree was evaluated by non-parametric bootstrapping (1,000 replications) with the thorough bootstrap option of RAxML under the general time-reversible (GTR) model with a gamma model (Γ) of the rate of heterogeneity for each of *trnL-F*, ITS and the combined sequence matrix.

### Chromosome preparations

Four species, *Boe. guileana*, *Boe. stolonifera* K.Y. Pan, *Be. baolianis* and *Be. tonkinensis* (Pellegr.) B.L. Burtt, were investigated cytologically. The plants for chromosome studies were collected from the field and cultivated in Gesneriad Conservation Center of China (GCCC). Actively growing root tips were collected and pretreated with 2 mM 8-hydroxyquinoline at 20 °C for about 2 hrs, then fixed with Farmer’s solution (absolute alcohol: glacial acetic acid 3:1) at 4 °C for about 2 hrs. After hydrolysis for 30 min in 1 M HCl at room temperature, followed by washing through several changes of distilled water, the roots were transferred to carbol fuchsin for about 2 hrs.

## Results

### Morphological affinities

**Figure 1.**
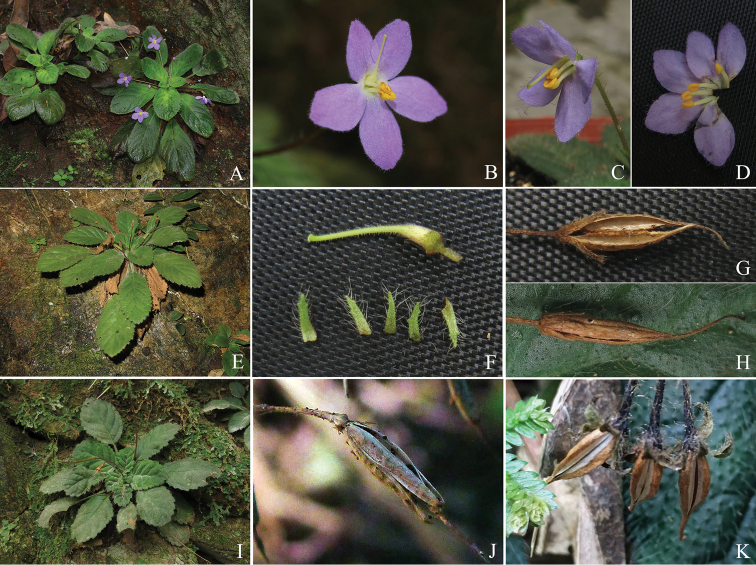
Photographs of *Oreocharis guileana* (**A–H**), *O. pilosopetiolata* (**I**), *Boeica ferruginea* (**J**) and *Beccarinda tonkinensis* (**K**). **A, E, I** habit **B, C** flower **D** opened corolla, showing stamens and staminodes **F** pistil **G, H, J, K** mature fruit.

**Figure 2.**
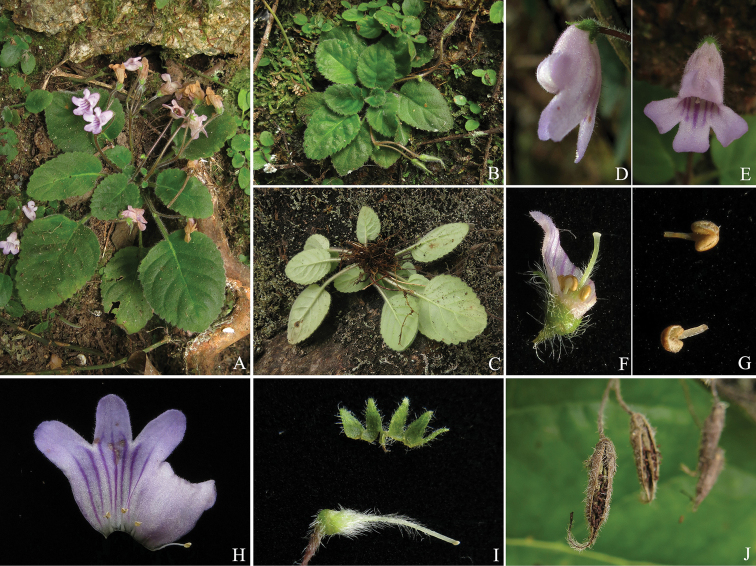
Photographs of *Oreocharis baolianis*. **A–C** Habit **D, E** flower **F, H** opened corolla, showing stamens and staminodes **G** anthers **I** pistil **J** mature fruit.

The results of morphological observation indicated that both of *Boe. guileana* (Fig. 1A–H) and *Bec. baolianis* (Fig. 2) should be deposited into the enlarged *Oreocharis*. Both of these two species possessed these characters: rosette herbaceous habit (Figs 1A, E, 2A–C), longitudinally dehiscent anther (Figs 1D, 2G) and loculicidal dehiscent capsule (Figs 1G, H, 2J), which showed distinct relationships to *Boeica* or *Beccarinda* and the equivalent characters of these two genera were discussed below. Our morphological comparisons showed *Boe. guileana* was similar to *Ore. pilosopetiolata* in the texture, shape and margin of leaf and the pubescence on most part of the plants. Nevertheless, *Bec. baolianis* showed a special combination of characters in the enlarged *Oreocharis*, such as small plant size, campanulate corolla and short filaments. The morphological affinities of *Bec. baolianis* are discussed below. Additionally, we amended or supplemented the description of some characters (such as dehiscent way of anther and fruit, corolla lobes margin and calyx) of these two species here and the detailed amended descriptions were given below.

### Matrix characteristics

The aligned *trnL-F* and ITS datasets were 966 and 824 characters long, thereof, 195 and 138 were parsimony-uninformative and 139 and 388 parsimony-informative characters, respectively. The combined dataset was 1790 characters long with 527 (29.4%) parsimony-informative characters. The incongruence length different (ILD) test showed no significant incongruence between the ITS and *trnL-F* (p = 0.53).

### Phylogenetic analyses

**Figure 3.**
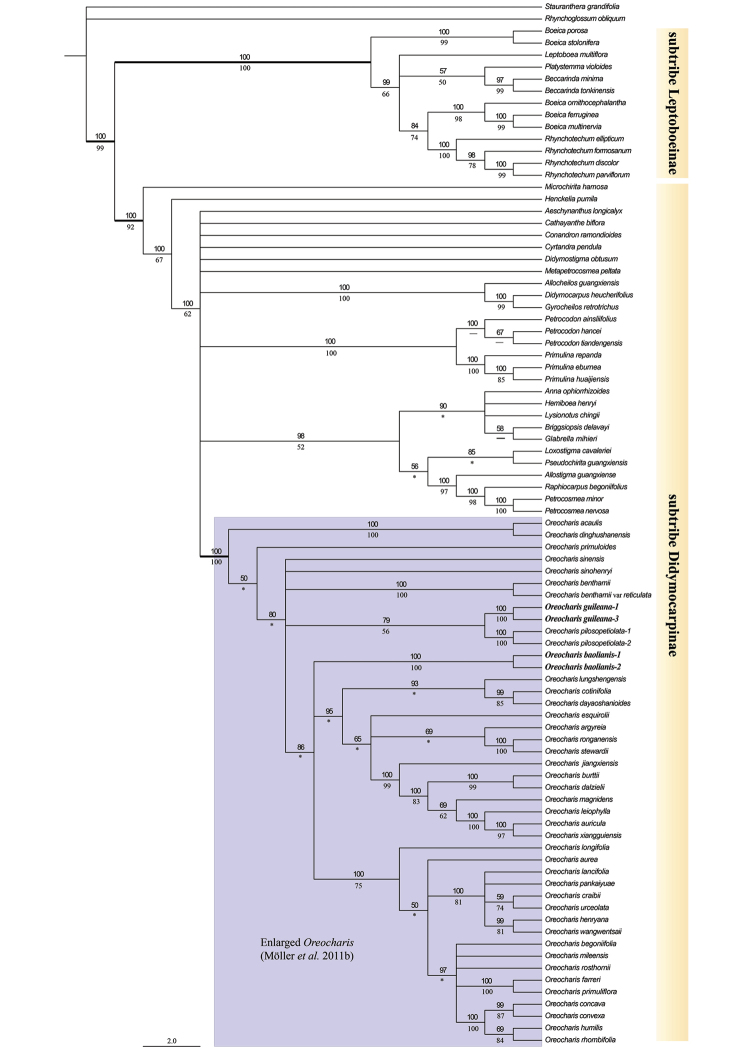
Bayesian (> 50%) tree resulting of the combined nuclear (ITS) and plastid (*trnL-F*) data matrices. Posterior probability (PP) from the BI analysis are indicated above branches and Bootstrap value (BS) from the ML analysis are indicated below. The asterisk indicates a BS < 50. The dash indicates the topological discordance between ML and Bayesian tree. The two species, *O. baolianis* and *O. guileana*, are highlighted in bold.

In the combined DNA sequence analysis, the BI tree was less resolved but congruent (virtually identical) where the ML branches received bootstrap support > 50% (Fig. 3). The phylogenetic relationships reconstructed from the concatenated matrix were generally congruent with those reported in Möller et al. (2011a, b). The subtribe Leptoboeinae (BS = 100%, PP = 100%) and Didymocarpinae (BS = 100%, PP = 92%) were both monophyletic with high support values. The enlarged *Oreocharis* formed a well-supported (BS = 100%, PP = 100%) monophyletic clade within the subtribe Didymocarpinae (Fig. 3). As expected, *Boe. guileana* and *Be. baolianis* fell into the enlarged *Oreocharis* clade (Fig. 3). Although with relatively low support (BS = 56%, PP = 79%), *Boe. guileana* is sister to *Oreocharis pilosopetiolata* in the current phylogeny (Fig. 3). However, the phylogenetical position of *Be. Baolianis* in the enlarged *Oreocharis* was difficult to discuss here. *Beccarinda baolianis*, together with two other larger clades, formed a polytomy in our phylogenetical analyses (Fig. 3).

### Chromosome cytology

**Figure 4.**
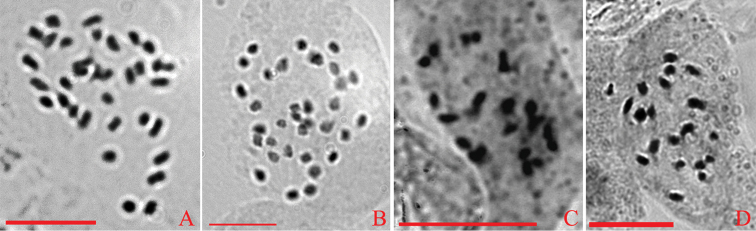
Somatic metaphase chromosome spreads of *Oreocharis guileana*, 2n = 34 (**A**), *O. baolianis*, 2n = 34 (**B**), *Beccarinda tonkinensis*, 2n = 20 (**C**) and *Boeica stolonifera*, 2n = 20 (**D**). Scale bar: 10 μm.

In the four investigated species, the somatic chromosomes were determined to be diploid with 2n = 34 in *Boe. guileana* (Fig. 4A) and *Be. baolianis* (Fig. 4B) and 2n = 20 in *Be. tonkinensis* (Fig. 4C) and *Boe. stolonifera* (Fig. 4D).

## Discussion

Traditionally, the classification of the Old World Gesneriaceae is heavily based on floral characters (e.g. Burtt 1977; Wang et al. 1998). However, recent phylogenetic studies reveal that the diversity of floral characters within this family has a complex evolutionary background, i.e. multiple convergences and parallel changes (Wang et al. 2010; Möller et al. 2011b; Weber et al 2011; Lu et al. 2017). Therefore, wrong generic placements have been made for some species based on the homoplasious floral traits. For example, symmetrical corolla was regarded as an original character in the Old World Gesneriaceae (Wang et al. 1998) and several genera have been established by this character such as *Bournea* Oliv. (Hooker 1893), *Tengia* Chun (Chun 1946) and *Thamnocharis* Wang (Wang 1981); but the recent phylogenetic studies indicate that the symmetrical corolla convergently evolve in different alliances of Old World Gesneriaceae (Wang et al. 2010) and all these three genera have been incorporated into other genera (Möller et al. 2011b; Weber et al. 2011). In the recent case of *Wentsaiboea tiandengensis* Yan Liu & B. Pan (Liu et al. 2010) and *Primulina guangxiensis* Yan Liu & W.B. Xu (Liu et al. 2011), molecular phylogenetical research reveals that these species belong to other genera as *Petrocodon tiandengensis* (Yan Liu & B. Pan) A. Weber & Mich. Möller (Weber et al. 2011) and *Petrocodon guangxiensis* (Yan Liu & W.B. Xu) W.B. Xu & K.F. Chung (Xu et al. 2014), respectively.

In the present study, we show a similar finding for *Boe. guileana* and *Be. baolianis*. We combine detailed morphological studies, cytological examinations and phylogenetic analyses to show that *Boe. guileana* and *Be. baolianis* are two species belonging to the enlarged *Oreocharis*. The morphological characters of these two species, i.e. growth habit, anther and fruit dehiscence are clearly distinct from its original genera. *Boeica guileana* and *Be. baolianis* are both rosette plants with inconspicuous rhizome (Figs 1A, E, 2A–C), which is distinctly different from most of other *Boeica* (caulescent habit; Burtt 1977; Wang et al. 1998; Weber and Skog 2007) and *Beccarinda* (long rhizomatous, caulescent or stoloniferous habit; Burtt 1955; Wang et al. 1998; Weber and Skog 2007) species. The anthers of *Boe. guileana* (Fig. 1D) and *Be. baolianis* (Fig. 2G) are longitudinally dehiscent which is different from other *Boeica* and *Beccarinda* species (poricidally- or transversely-dehiscent anthers; Burtt 1955, 1977; Wang et al. 1998; Weber and Skog 2007). In addition, fruits of both *Boe. guileana* (Fig. 1G, H) and *Be. baolianis* (Fig. 2J) are loculicidally dehiscent, which is a character compatible to the enlarged *Oreocharis* (Möller et al. 2011b). In other *Boeica* species, fruits dehisce septicidally or loculicidally (Fig. 1J; Weber and Skog 2007) and in other *Beccarinda* species, the fruits dehisce septicidally (Fig. 1I; Weber and Skog 2007). The somatic chromosome numbers of *Boe. guileana* and *Be. baolianis* were determined as diploid with 2n = 34 (Fig. 4A, B), which is consistent with previous findings for species within the enlarged *Oreocharis* (Ratter 1963; Ratter and Prentice 1964; Fussell 1958; Wang and Gu 1999; Lu et al. 2002; Zhou et al. 2003; Tan et al. 2011). The somatic chromosome numbers of *Boeica* and *Beccarinda* species studied here were 2n = 20 (Fig. 4C–D) which is also consistent with previous studies (Ratter and Prentice 1964), except one case of *Boe. brachyandra* was recorded as 2n = +/-22 (Kiehn et al. 1998). Ultimately, our phylogenetic analyses prove with high support rate (Fig. 3) that *Boe. guileana* and *Be. baolianis* belong to the enlarged *Oreocharis* clade. Additionally, the further phylogenetic analysis, based on some additional plastid sequences, revealed the above relationship (pers. Yu-Min Shui).

After incorporation of ten additional genera, the genus *Oreocharis* becomes a large group with high flower diversity. Flowers of this group are extremely variable in corolla shape, colour, number of stamens and anthers being free or fused (Möller et al. 2011b). However, the corolla shapes and the filament length of *Boe. guileana* (filament 3–4 mm long) and *Be. baolianis* (filament 2.5–3 mm long) are rather uncommon in the enlarged *Oreocharis*. Therefore, the addition of these two species further enriches the diversity of flower morphology within the genus *Oreocharis*.

### Taxonomic treatment

#### Oreocharis guileana

Taxon classificationPlantaePasseriformesParamythiidae

(B.L. Burtt) Li.H.Yang & F.Wen comb. nov.

33A23094-067C-5C24-BD22-E66DA2193FF3

urn:lsid:ipni.org:names:77211180-1

Figure 1A–H

≡Boeica guileana B.L. Burtt (1977: 371).

##### Type.

China. Hong Kong: New territories, Ma On Shan, 690 m alt., ravine in montane forest, on rocks in humid shade, July 1974, Guile; culture in R.B.G. Edinb. 1976, *C.8467* (holotype: E!).

##### Amended description.

Perennial herbs with inconspicuous rhizome. Leaves in basal rosette, 8–22; petiole 1–3 cm long, with densely white villous; leaf blade elliptic to ovate or obovate, 2–6 × 1.1–3.2 cm, white villous and pubescent on both sides, abaxially more densely villous along veins, base cuneate, margin serrate, apex acute to rounded; lateral veins 4–6 on each side of midrib. Cymes 1–5, axillary, 1–4-flowered. Peduncles 2.5–5.5 cm long, with brown villous; bracts 2, lanceolate, 2.5–3 × ca. 1 mm, margins entire, outside brown villous; pedicel 1–2 cm long, with brown villous. Calyx 5-lobed near base, lobes equal, lanceolate to linear, 3–5 × ca. 1 mm, outside covered by brown villous, margin entire. Corolla blue-purple, outside with sparely-brown villous; tube short and not swollen, 1.5–2.5 mm long; adaxial lip 2-lobed near base, lobes obovate-oblong, margin entire or slightly erose, apex obtuse, 5–7 × 4–5 mm; abaxial lip 3-lobed near base, lobes obovate-oblong, margin entire or slightly erose, apex obtuse, 9–12 × 4.5–6 mm. Stamens 4, adnate to corolla base, filaments linear, with sparsely pubescent, adaxial stamens ca. 3 mm long, abaxial stamens ca. 4 mm long, anthers dorsifixed, free, ovate-oblong, dehiscing longitudinally, glabrescent; staminode absent; disc not obvious; pistil 8–12 mm long; ovary conical, 2–3.5 mm, with densely-white villous; style 6–8 mm long, with pubescent. Stigma 1, disc-shaped. Capsule ca. 1.5 cm long, villous, dehiscing loculicidally to base, initially on one side, valves 2, capsule straight in relation to pedicel, not twisted.

##### Distribution and habitat.

**Figure 5.**
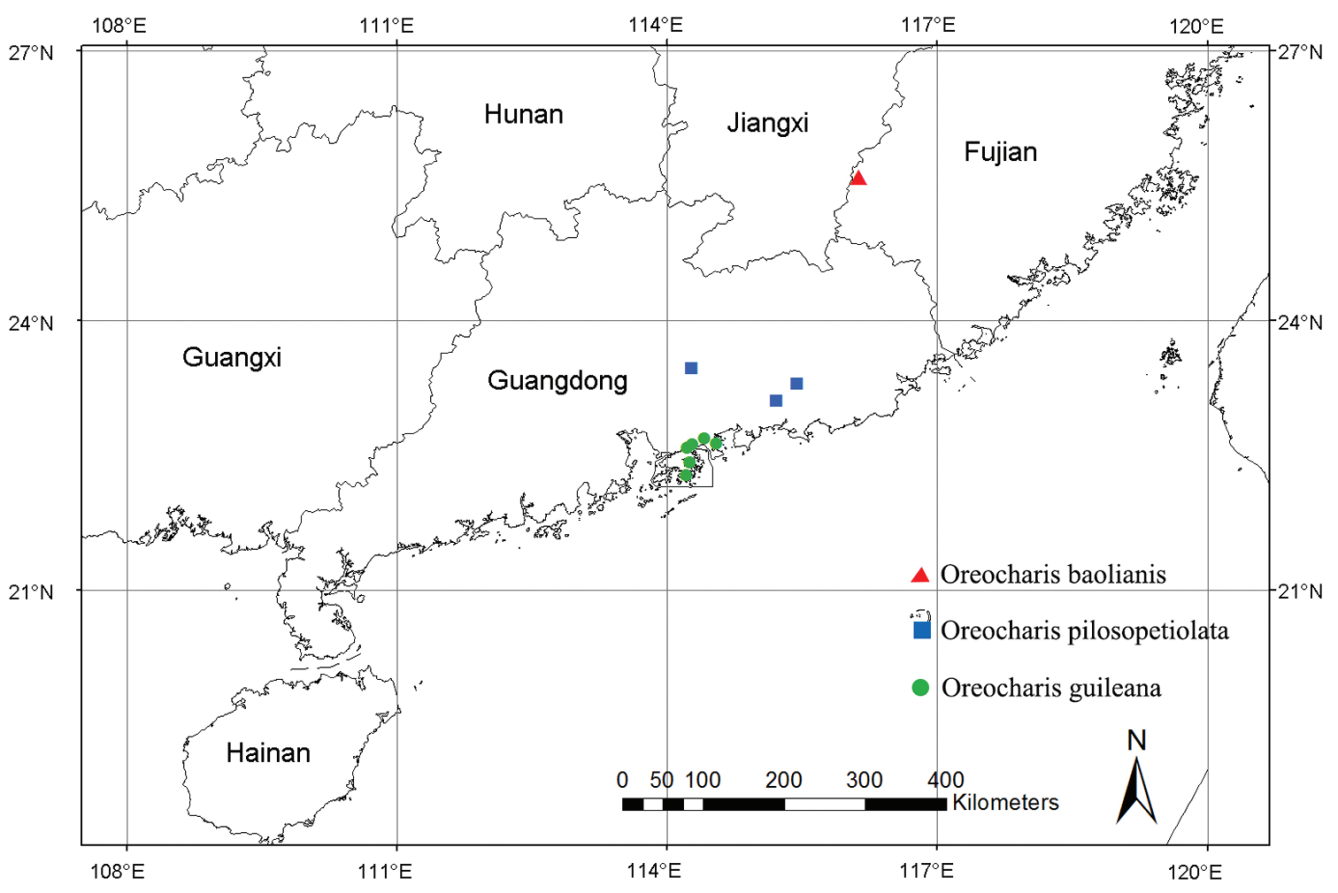
Geographical distribution of *Oreocharis guileana* (green dot), *O. baolianis* (red triangle) and *O. pilosopetiolata* (blue square).

*Oreocharis guileana* was once recognised as an endemic species and only recorded at Ma On Shan, Hong Kong. However, recent field works reveal that it can be found from several sites in Shenzhen, such as Wu Tong Shan, Pai Ya Shan, Tian Xin Shan, Dakeng reservoir and Xigong village (Fig. 5). The plants grow on moist rock surfaces under evergreen broad-leaved forests, at an altitude of 300–900 m.

##### Note.

Burtt (1977) thought that *Oreocharis guileana* is morphologically similar to *Boe. brachyandra* and *Boe. nutans* by its rosette habit, short corolla tube and spreading limb. However, as mentioned above, the currently taxonomic status of these two *Boeica* species is doubtful and need further studies to confirm it. Additionally, the distinctively disjunctive distribution between *Oreocharis guileana* and *Boe. brachyandra* and *Boe. nutans* may indicate a distant relationship between them (*Oreocharis guileana* is a species occurring in S China, but *Boe. brachyandra* and *Boe. nutans* are distributed on the Malayan Peninsula). Although with low support, our phylogenetic results show that *Oreocharis guileana* and *O. pilosopetiolata* have a sister relationship (Fig. 3). These two species have similar leaf blade (size, shape, texture and margin) and indumentum on most of organs and it is difficult to distinguish these two species without flowers. Additionally, the related adjacent geographic distribution also indicates the close relationship between these two species (Fig. 5).

##### Additional specimens examined.

China. Shenzhen Special Economic Zone, Pingshan District, Tian Xin Shan, alt. 300 m, 22 May 2017, 114°25'E, 22°41'N, *L.H. Yang & F. Wen YLH383* (IBSC!); in the same place, alt. 300 m, 20 October 2005, *S.Z. Zhang et al. 4658* (SZG!); 20 April 2005, *S.Z. Zhang et al. 0384* (SZG!); 5 November 2004, *S.Z. Zhang et al. SCAUF490* (SZG!), *SCAUF491* (SZG!), *SCAUF583* (SZG!); Longgang District, Pai Ya Shan, alt. 600 m, 25 October 2006, *G.D. Wang et al. 6896* (SZG!); 13 October 2005, *S.Z. Zhang et al. 4533* (SZG!); 17 December 2005, *S.Z. Zhang et al. 2144* (SZG!); 8 June 2005, *S.Z. Zhang et al. 2340* (SZG!); Xi Chong, Xigong village, 8 November 2004, *S.Z. Zhang et al. SCAUF469* (SZG!), *SCAUF470* (SZG!); Luohu District, Wu Tong Shan, alt. 900 m, 7 October 2005, *S.Z. Zhang et al. 4288* (SZG!); 23 March 2005, *Team of Flora of Shenzhen 013556* (SZG!); Longgang District, Dakeng reservoir, 16 July 2005, *S.Z. Zhang et al. 2932* (SZG!).

#### Oreocharis baolianis

Taxon classificationPlantaePasseriformesParamythiidae

(Q.W. Lin) Li.H.Yang & M.Kang comb. nov.

657D1074-748E-55EE-B3FB-27C7FBC93370

urn:lsid:ipni.org:names:77211181-1

## Supplementary Material

XML Treatment for Oreocharis guileana

XML Treatment for Oreocharis baolianis

## Supplementary materials

Supplementary material 1 Taxon, voucher information and GenBank accession number of the samples used in the phylogenetic analyses. Data type phyogenetic https://binary.pensoft.net/file/445572This dataset is made available under the Open Database License (http://opendatacommons.org/licenses/odbl/1.0/). The Open Database License (ODbL) is a license agreement intended to allow users to freely share, modify, and use this Dataset while maintaining this same freedom for others, provided that the original source and author(s) are credited.Lihua Yang, Fang Wen, Hanghui Kong, Zhixia Sun, Lanying Su, Ming Kang
